# Study of cardiovascular disease prediction model based on random forest in eastern China

**DOI:** 10.1038/s41598-020-62133-5

**Published:** 2020-03-23

**Authors:** Li Yang, Haibin Wu, Xiaoqing Jin, Pinpin Zheng, Shiyun Hu, Xiaoling Xu, Wei Yu, Jing Yan

**Affiliations:** 1 Zhejiang Provincial Center for Cardiovascular Disease Control and Prevention, Zhejiang Hospital, Hangzhou, 310013 China; 2 Ewell Technology Co., Ltd, Tower D of Oriental Communication Technology City, Hangzhou, 310000 China; 3 Chinese Acupuncture Department, Zhejiang Hospital, Hangzhou, 310013 China; 4 Key Laboratory of Public Health Safety, Ministry of Education, Health Communication Institute, Fudan University, 138 Yixueyuan Road, Shanghai, 200032 China

**Keywords:** Disease prevention, Public health, Risk factors

## Abstract

Cardiovascular disease (CVD) is the leading cause of death worldwide and a major public health concern. CVD prediction is one of the most effective measures for CVD control. In this study, 29930 subjects with high-risk of CVD were selected from 101056 people in 2014, regular follow-up was conducted using electronic health record system. Logistic regression analysis showed that nearly 30 indicators were related to CVD, including male, old age, family income, smoking, drinking, obesity, excessive waist circumference, abnormal cholesterol, abnormal low-density lipoprotein, abnormal fasting blood glucose and else. Several methods were used to build prediction model including multivariate regression model, classification and regression tree (CART), Naïve Bayes, Bagged trees, Ada Boost and Random Forest. We used the multivariate regression model as a benchmark for performance evaluation (Area under the curve, AUC = 0.7143). The results showed that the Random Forest was superior to other methods with an AUC of 0.787 and achieved a significant improvement over the benchmark. We provided a CVD prediction model for 3-year risk assessment of CVD. It was based on a large population with high risk of CVD in eastern China using Random Forest algorithm, which would provide reference for the work of CVD prediction and treatment in China.

## Introduction

Cardiovascular disease (CVD) is a series of diseases involving the circulatory system, including angina pectoris, myocardial infarction, coronary heart disease, heart failure, arrhythmia and else, which is generally related to atherosclerosis. With the social economy development, the population aging and the urbanization acceleration in China, some changes have taken place in national lifestyles, which leading to a rise of CVD prevalence. In 2016, there were more than 290 million cases of CVD in China, and 4.344 million deaths from it, including 2.098 million deaths from stroke and 1.736 million deaths from coronary heart disease, which bringing heavy social and economic burden^1^. CVD is a disease that can be prevented and controlled, and early intervention can effectively control its progress^2^.

In recent years, many achievements have been made in the study of CVD risk prediction model, but the effect of epidemiological risk factors and biomarkers may be different in different populations, the CVD model has certain population specificity. In addition, there has been no study on CVD risk prediction model based on large cohort population in eastern China. At the same time, a large number of the existing CVD prediction models use multivariable regression method to build prediction models in a linear fashion, but it generally exhibit modest predictive performance, especially for certain sub-populations^3,4^. Machine learning (ML) such as random forest (RF) can improve the performance of risk predictions by exploiting large data repositories to identify novel risk predictors and more complex interactions between them^3^.

In this study, we conducted a CVD prediction model research based on a specific culture, lifestyle, behavior and genetic background in eastern China. From September 2014 to December 2016, a cohort of 25231 subjects with high-risk CVD were selected from 101056 people in Zhejiang province. Cardiovascular events were collected through regular follow-up using the electronic health record (EHR) system, and a CVD prediction model for 3-year risk assessment of CVD was constructed using the RF algorithm based on classification and regression tree (CART).

## Methods

### Study design and study population

The project was one of the centers of the national high-risk screening program, and its design and population screening had been published in other journals^5^. The large program was patient-centered evaluative assessment of cardiac events including 1.7 million persons in China, and it is a population-cantered national screening initiative to detect populations at high risk of CVD.

Our study was conducted in 6 geographically defined regions of Zhejiang province in China that began from Sept 15, 2014 and has continued by now. Subjects identified as being at high risk of CVD in community health center were moved to hospital to receive further assessment and follow-up care. Participants are considered at high risk of CVD if they meet at least one of criteria. The criteria are adapted from WHO guidelines for the assessment and management of cardiovascular risk^6^. Inclusion criteria: subjects aged ≥35 years, living in selected community, with normal cognitive function, high risk of CVD, and able to cooperate with investigation and relevant referral. Exclusion criteria: subjects with new cardiovascular and cerebrovascular disease events within half a year, including angina pectoris, stroke, acute myocardial infarction, coronary heart disease, heart failure, arrhythmia and else, subjects with severe dementia, severe liver and kidney dysfunction, subjects with acute critical illness, incoordination. All participants provided written informed consent.

### Data collection and follow-up

From 2014 to 2016, a screening was conducted among more than 100,000 residents in Zhejiang province, mainly through questionnaire survey, physical examination, laboratory examination and else, to understand risk factors related CVD, evaluate CVD risk, determine high-risk CVD subjects, and a cohort was established conducting follow-up. Follow-up was conducted once a year to collect socio-economic information, and blood samples were collected every two years.

For each participant, blood pressure, lipid and blood glucose levels, height, and weight were measured at the initial screening. Blood pressure (BP) was measured twice on the right upper arm after 5 minutes of rest in a seated position with a standardized electronic blood pressure monitor (Omron HEM-7430)^7^. If the difference between the 2 systolic blood pressure (SBP) readings was greater than 10 mmHg, a third measurement was obtained and the average of the last 2 readings was used. Participants were required to wear light clothes, no shoes while being measured for height and weight^8^.

Trained nurses conducted standardized interviews among the participants to collect information on sociodemographic status (ethnicity, education level, occupation, marital status, annual household income, medical insurance status and so on), lifestyle (smoking and alcohol use), medical history, and medication use. The questionnaire is designed by Fuwai Hospital.

### Ethics approval

The protocol of this study was approved by the National Center for Cardiovascular Disease (NCCD) and the Medical Ethics Committee of Zhejiang Hospital. The participants were informed about the objectives and methods of the study. They were informed that their participation was totally voluntary and that they could withdraw from the study at any time without citing any reason. Written and signed or thumb printed informed consent was obtained from those who agreed to participate, or from their guardians. The methods used in this research were carried out in accordance with the approved guidelines. Trial registration number: NCT02536456.

### Definition

The definitions of variables were on the basis of Chinese recommendations from the Working Group on Obesity and Chinese guidelines on prevention and treatment of dyslipidemia in adults^9–11^. The detailed information of definitions was shown in Table 1.

**Table 1:** Definition of some variables.

Variables	Definition
Overweight	BMI ≥24 kg/m^2^ and <28 kg/m^2^
Obesity	BMI ≥28 kg/m^2^
Waistline is large	waistline ≥85 cm for the male or waistline ≥80 cm for the female
Smokers	Subjects who smoked one cigarette or more per day for over 6 months
Abnormal TG	TG ≥ 2.3 mmol/L
Abnormal TC	TC ≥ 6.2 mmol/L
Abnormal LDL	FLDL ≥ 4.1 mmol/L
Abnormal HDL	HDL < 1.0 mmol/L
Abnormal FPG	FBG ≥ 6.2 mmol/L

BMI, body mass index; FBG, Fasting plasma glucose; TC, Total cholesterol; TG, triglycerides; LDL, Low density lipoprotein; HDL, High density lipoprotein.

### Statistical analysis

All statistical analysis was conducted by using R version 3.2.5 (R Foundation for Statistical Computing, Vienna, Austria). Enumeration data were expressed as percentages (%) and were compared using the χ2 test, and data were analyzed using Yates’s continuity correction or Fisher’s exact probability test as necessary. Univariate logistic regression analysis was performed to screen related risk factors of CVD. We found the point on the ROC curve that is closest (i.e., the shortest distance) to the perfect model (with 100% sensitivity and 100% specificity), which was associated with the upper left corner of the plot^12^. Then we used the confusion matrix to calculate the sensitivity and specificity of every important risk factor.

$$Sensitivity=\frac{\#\,samples\,with\,the\,event\,and\,predicted\,to\,have\,the\,event}{\#\,samples\,having\,the\,event}$$

$$Specificity=\frac{\#\,samples\,without\,the\,event\,and\,predicted\,as\,nonevents}{\#\,samples\,without\,the\,event}$$

The discrimination ability of the model was evaluated by using receiver operator characteristic (ROC) curve analysis. The AUC > 0.5 indicated better predictive values, the closer the AUC to 1, the better the model performance. Model calibration was checked by using the Hosmer–Lemeshow goodness-of-fit test to determine whether chance could explain the difference between the predicted and the observed event rate^13^. *P* value of less than 0.05 indicated a statistically significant difference.

**Figure 1:**
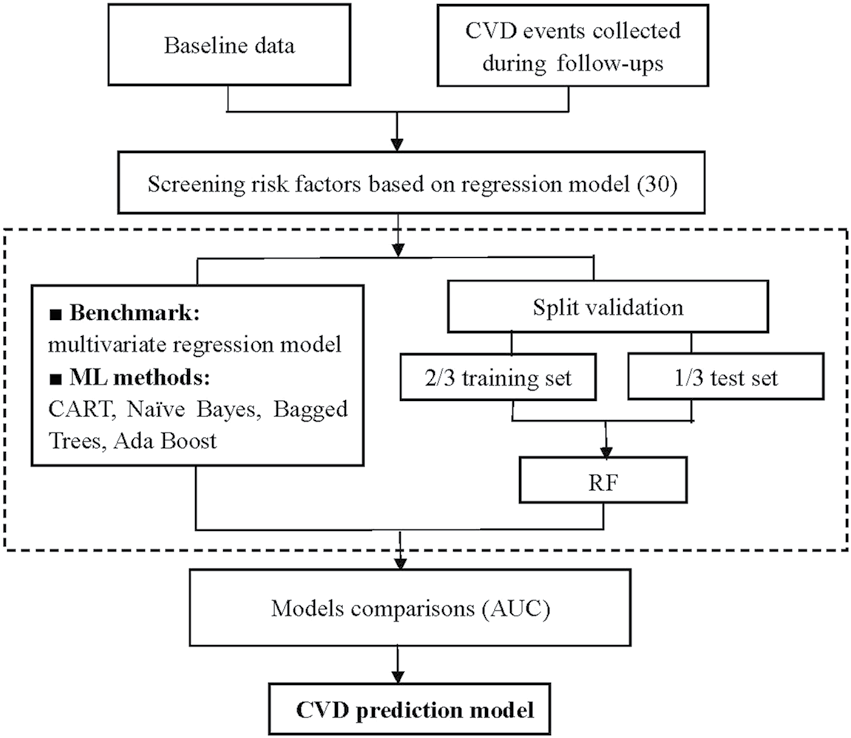
An illustrative schematic for CVD prediction model.

We established a CVD risk prediction model suitable for population in Zhejiang province based on RF. Several methods were used to be compared with RF, including multivariate regression model, classification and regression tree (CART), Naïve Bayes, Bagged trees and Ada Boost. And the multivariate regression model was used as a benchmark for performance evaluation (AUC=0.7143). The illustrative schematic for CVD prediction model conduction was shown in Fig. 1.

The decision tree model was a tree structure composed of root node, branch node and leaf node, which reflected the mapping relationship between features and tags. Information gain was used to measure the influence of a certain feature on the classification result. Suppose the sample data set was D, which contained n types of data, and the sample proportion of the ith type of data in the total data set was *p*_*i*_, and the information entropy of the data set D was ${\rm{Info}}({\rm{D}})=-\,{\sum }_{i=1}^{n}\,{p}_{i}lo{g}_{2}({p}_{i})$. We selected feature A as the decision feature of the decision tree, divided data set D into k parts, and then the conditional entropy of feature A to data set D was $\,{\rm{Info}}(D|A)={\sum }_{j=1}^{k}\frac{{D}_{j}|}{|{D}_{i}}\times {\rm{Info}}({D}_{j})$. According to the definition of information gain, it could be known that after the action of feature A on data set D, the decrease value of information entropy was ${\rm{Gain}}({\rm{A}})={\rm{Info}}({\rm{D}})-{\rm{Inf}}{o}_{A}(D)$. When Gain (A) reached the maximum value, this feature was the most appropriate node selection of the decision tree^12^.

#### CART

It was an implementation of decision tree. Generally, there were three implementations of decision tree, namely ID3 algorithm, CART algorithm and C4.5 algorithm. CART algorithm was a binary recursive segmentation technology, which divided the current sample into two sub-samples, so that each generated non-leaf node had two branches. Therefore, the decision tree generated by CART algorithm was a binary tree with simple structure. Since CART algorithm was a binary tree, it could only be “yes” or “no” in the decision of each step. Even if a feature had multiple values, it would divide the data into two parts. CART algorithm was mainly divided into two steps: recursively divide the samples for the tree building process, pruning with validation data^12^.

RF was an ensemble learning method based on decision tree. It adopted the re-sampling technique of bootstrap to repeatedly randomly select b samples from the original training sample set of N as the training set and the remaining samples as the test set. We adopted the method of random sampling in proportion, and generated a new training sample set from Linan (4533), Zhuji (3542), Anji population (4990). We randomly selected m feature sets from each training self-help sample, and then generated B decision trees according to the self-help sample set. When the decision tree was split, the optimal feature set was selected from m features. B decision trees constituted the random forest, and the classification results of new data were determined by the number of votes in the decision tree.

#### Naïve bayes

Bayes’ Rule answers the question “based on the predictors that we have observed, what is the probability that the outcome is class Ce?” More mathematically, let Y be the class variable and X represent the collection of predictor variables. We were trying to estimate ${P}_{r}[Y={C}_{e}|X]$, which was “given X, what is the probability that the outcome is the eth class?” Bayes’ Rule provided the machinery to answer this: ${P}_{r}[Y={C}_{e}|X]=\frac{{P}_{r}[Y]{P}_{r}[X|Y={C}_{e}]}{{P}_{r}[X]}$, ${P}_{r}[Y={C}_{e}|X]$ was typically referred to as the posterior probability of the class^12^.

#### Bagged trees

Bagging for classification was a simple modification to bagging for regression. Specifically, the regression tree was replaced with an unpruned classification tree for modeling C classes. Since each model had equal weight in the ensemble, each model could be thought of as casting a vote for the class it thought the new sample belonged to. The total number of votes within each class were then divided by the total number of models in the ensemble (M) to produce a predicted probability vector for the sample. The new sample was then classified into the group that had the most votes, and therefore the highest probability^12^.

#### Ada boost

To summarize the algorithm, AdaBoost generated a sequence of weak classifiers, where at each iteration the algorithm found the best classifier based on the current sample weights. Samples that were incorrectly classified in the kth iteration received more weight in the (k + 1)st iteration, while samples that were correctly classified received less weight in the subsequent iteration. At each iteration, a stage weight was computed based on the error rate at that iteration. The overall sequence of weighted classifiers was combined into an ensemble and had a strong potential to classify better than any of the individual classifiers.

#### Framingham risk score

The Framingham score is based on 7 core risk factors including of gender, age, systolic blood pressure, treatment for hypertension, smoking status, history of diabetes, and BMI. All of those variables were complete for the participants in the extracted cohort. The number of imputed datasets was selected via cross-validation.

## Results

### Baseline situation and related risk factors of CVD

From September 2014 to December 2016, 29930 participants with high-risk of CVD were selected from 101056 people in Zhejiang province, and a cohort of population with high-risk CVD was established. The network management system was used for regular follow-up and blood sample collection. There were 25231 subjects with 976 cardiovascular disease events in the cohort until the end of 2016. A total of 15.7% of participants were lost to follow-up.

The results showed that the incidence of CVD in this cohort was about 3.9% in three years and was expected to increase to about 5% by 2021. Overall, the mean (SD) age of participants at baseline was 58.1 (10.6) years, and 13528 (53.62%) were female, 23269 (92.22%) were married, 1495 (5.93%) had high school education or above, 9676 (38.35%) were overweight, 4420 (17.52%) were obesity, 11718 (46.44%) had hypertension and 2256 (8.94%) had diabetes.

Using univariate logistic regression analysis, combined with the professional knowledge, we screened the important variables of CVD. The analysis revealed that nearly 30 indicators including male, older age, family income, smoking, excessive drinking, obesity, large waistline, abnormal cholesterol, low HDL-C, abnormal FPG, low ability to action were related to CVD (Supplementary Table 1).

According to the initial screening variables, the univariate ROC curve was used to analyze the prediction ability of continuous variables. The results of univariable ROC analysis indicated important evaluation parameters including of AUC, threshold, sensitivity, specificity of important prediction variables of CVD (Table 2).

**Table 2:** Performance of related risk factors of CVD.

Variables	Threshold	Specificity	Sensitivity	AUC	95%CI
Age	61.50	0.7134	0.5962	0.6916	0.6515–0.7316
SBP	156.75	0.7261	0.5735	0.6478	0.6075–0.688
DBP	88.25	0.7006	0.5493	0.6525	0.6138–0.6912
FBG	6.36	0.6433	0.3997	0.5032	0.4565–0.55
HDL	1.39	0.5350	0.5023	0.5081	0.4621–0.5541
LDL	2.55	0.5924	0.4952	0.5691	0.525–0.6132
TC	4.31	0.4777	0.6556	0.5757	0.5296–0.6218
TG	1.35	0.5350	0.5123	0.5194	0.4752–0.5635
BMI	24.89	0.6433	0.4774	0.5596	0.5153–0.604
Waistline	84.05	0.5287	0.5056	0.5215	0.4756–0.5675
HR	72.25	0.4904	0.5776	0.5392	0.4903–0.5881
PEF	291.00	0.5096	0.5989	0.5659	0.5184–0.6134

AUC, Area under the curve; SBP, systolic blood pressure; DBP, diastolic blood pressure; FBG, Fasting plasma glucose; HDL, High density lipoprotein; LDL, Low density lipoprotein; TC, Total cholesterol; TG, triglycerides; BMI, body mass index; HR, Heart rate; PEF, peak expiratory flow. Uni-variate ROC curve was used to analyze the prediction ability of key continuous variables.

### CVD prediction model development and its performance

**Figure 2:**
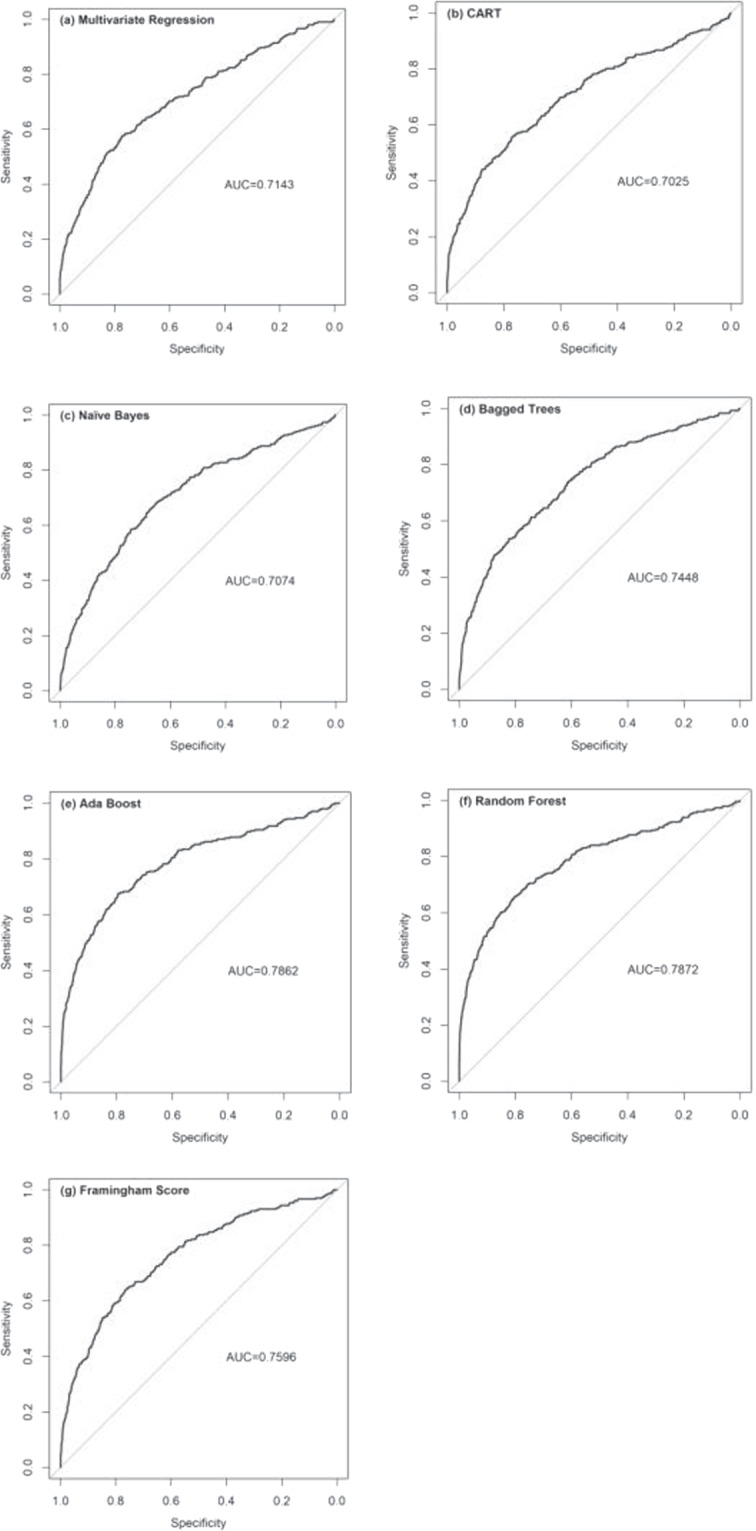
ROC curves of prediction models for CVD. (**a**) ROC curve of Multivariate Regression model for CVD. (**b**) ROC curve of CART model for CVD. (**c**) ROC curve of Naïve Bayes model for CVD. (**d**) ROC curve of Bagged Trees model for CVD. (**e**) ROC curve of Ada Boost model for CVD. (**f**) ROC curve of Random Forest model for CVD. (**g**) ROC curve of Framingham Score model for CVD.

The important variables screened by univariate logistic regression model were used to build the multivariate prediction model for CVD based on random forest algorithm. We used split validation method that two-thirds of the samples were randomly selected as the training set, and the remaining as the test set. Meanwhile, we tried to reduce the difference between the positive rate in the training set and the one in the test set. In the training set, the random forest algorithm was used to establish the training model, and then the test set was used for prediction. The confusion matrix was shown in Table 3. Finally, the AUC was used to evaluate the prediction ability of the model that was 0.7871 (Fig. 2). The Hosmer-Lemeshow test was used for Measuring Calibration. The chance could explain the difference between the predicted and the observed event rate (χ2 = 10.31, *P* = 0.2423).

**Table 3:** Confusion matrix based on random forest algorithm.

Predicted	Observed	
	Event	Nonevent
Event	281	1784
Nonevent	21	6324

### Comparisons of prediction models using different algorithms

The prediction accuracies of the different models under consideration were shown in Table 4. We used the multivariate regression model as a benchmark for performance evaluation (AUC=0.7143). 5 machine learning models including CART, Naïve Bayes, Bagged Trees, Ada Boost and Random Forest were conducted to be compared to the baseline model. Some important experimental settings included that we set 500 trees in the RF and Ada Boost algorithm, respectively, and we set 30 trees of every bad in the Bagged Trees algorithm. The remaining parameters are set to default values. The results showed that the RF achieved a significant improvement (AUC change was +7.29%) over the benchmark, which was superior to other models including the Framingham Score (Table 4) (Fig. 2).

**Table 4:** Performance of prediction models under consideration.

Model	AUC	AUC Change
Multivariate Regression	0.7143	Benchmark
CART	0.7025	−1.18%
Naïve Bayes	0.7074	−0.69%
Bagged Trees	0.7448	3.05%
Ada Boost	0.7862	7.19%
Random Forest	0.7872	7.29%
Framingham Score	0.7596	4.53%

AUC, Area under the curve; CART, Classification and Regression Tree.

## Discussion

CVD is the leading cause of death worldwide and a major public health concern. Its risk assessment is crucial to many existing treatment and control guidelines^14–16^. Although the effect of primary health care in controlling CVD has been demonstrated before, controversies still exist on the benefits of applying risk prediction compared with those of the risk factor approach in population-based interventions^17,18^.

The preliminary survey results of our research group showed that many epidemiological factors were closely related to the occurrence of CVD, including old age, male, living alone, rural area, low education level, high BMI, large waist circumference, family history and else^9^. In the present study, data of a cohort of 25231 people with high risk of CVD and with 3 years’ follow-up were analyzed, and the results showed that nearly 30 indicators including of male, older age, smoking, excessive drinking, obesity, large waistline, abnormal TC, low HDL, abnormal FBG and low ability to action were related to CVD.

CVD prediction was one of the most effective measures for CVD control. There were many successful CVD prediction models in the world. The Framingham study put forward the concept of CVD “risk factors” for the first time^19^, mainly including age, gender, family history, high blood pressure. In the PCE model recommended by ACC/AHA of the United States, the AUC was 0.713 (African American male) − 0.818 (African American female)^20^. In addition, there were ABC-CHD model (C-index was 0.81), CVDPoRT model, Q-risk score model and so on^21–23^.

Wang Y *et al*. developed the lifelong risk assessment model of CVD and stroke in China^24^. Yang X *et al*. made use of large sample cohort data in prediction for ASCVD Risk in China to establish a China-PAR model for 10-year Risk and lifetime Risk assessment of CVD, and proposed a risk stratification standard suitable for Chinese people, C-statistics reached 0.794 (95% CI, 0.775–0.814) (for male) and 0.811 (95% CI, 0.787–0.835) (for female)^25^. In our study, the CVD prediction model for 3-year risk with an AUC of 0.787 for all, 0.823 for male and 0.675 for female.

As a consequence of the major changes in rates of CVD events internationally in the past few decades^26,27^ and the substantial changes in preventive treatments^28^, most published CVD risk prediction equations are now likely to be out-of-date because they are based largely on older cohorts^29^ such as the 2013 American College of Cardiology/American Heart Association PCEs^30^. Median predicted 5-year CVD risk using new PREDICT equations was only 2.3% in women and 3.2% in men, and so for the PCEs to markedly overestimate CVD risk was not surprising. Māori, Pacific, and Indian patients with high deprivation scores had predicted CVD risks that were twice as high as those of European or Chinese patients with low deprivation scores^26^.

CVD prediction models improved as more mathematical models been used in the prediction in recent years. ML played an increasingly important role in classification prediction problems. Previous studies had shown that ML had relatively accurate results in classification problems of epidemiological data. RF was an Ensemble Learning method based on decision tree. It adopted the resampling technique of bootstrap and selected feature sets by random sampling and random selection. It was not easy to produce overfitting phenomenon and had good anti-noise ability. The established model was robust and could deal with nonlinear problems. In addition, RF could deal with the problem of certain data loss, and could give the important score of each characteristic variable while classifying, according to which the variables that played an important role in classification could be screened out. The results of this study also showed that the RF achieved a significant improvement over the benchmark of multivariate regression model, and was superior to other ML models including CART, Naïve Bayes, Bagged Trees, Ada Boost.

There were several strengths of this study. Firstly, using the method of RF, a CVD risk prediction model that suitable for population in Zhejiang province of China was established with an AUC of 0.7871, which was one of the first CVD prediction models for large population in eastern China. Secondly, Random Forest used here had the advantages that unlike most ML algorithms, it could accept dirty data, and unlike some traditional regression models, it also could model nonlinear relations and accept both regression and classification problems at meanwhile.

Despite these strengths, there were several limitations should be addressed. The main limitation of the study was that it lacked external validation. ML could be deemed as internal validation to some extent since it consisted of multiple data-oriented analyses through randomly splitting the data repeatedly. And the validation and optimization of current model needed to be performed in future study. Otherwise, we did not collect information on participants’ attitudes and knowledge regarding control of high CVD risk, primary care physicians’ assessment of CVD risk, or medical recommendations. Though 30000 patients were a large cohort for traditional methodologies, it might not be big enough for training a RF model. More data could offer better AUC results. And the follow-up time of 3 years was relatively short. Finally, the dataset used in this research was imbalanced, applying techniques to handle imbalanced datasets should be performed in the future work.

## Conclusion

We provided a CVD prediction model for 3-year risk assessment of CVD, which achieved a significant improvement over the benchmark of multivariate regression model, and was superior to other ML models including CART, Naïve Bayes, Bagged Trees, Ada Boost. It was based on a large population with high risk of CVD in eastern China using Random Forest algorithm, which would provide reference for the work of CVD prediction and treatment in China. Further population-based studies of the CVD prediction model proposed in this study with more population, longer follow up time, covering more places in China with external validation are needed.

## Supplementary Information

Supplementary table.
