# Supplementary material for: Study of cardiovascular disease prediction model based on random forest in eastern China
Source: Sci Rep. 2020 Mar 23;10:5245. doi: 10.1038/s41598-020-62133-5 (PMC7090086; doi:10.1038/s41598-020-62133-5)
Supplement: Supplementary file 1 — Supplementary table. [file 41598_2020_62133_MOESM1_ESM.pdf]

Study of cardiovascular disease prediction model based on random forest in eastern China

YANG Li[1,4,#], WU Haibin[2,#], JIN Xiaoqing[3], ZHENG Pinpin[4], HU Shiyun[1], XU Xiaoling[1], YU Wei[1], YAN Jing[1,*]

Variables	Overall (n=25231)	CVD (n=976)	Normal (n=24255)	χ2	*P*	OR (95%CI)
Gender						
Female	13528 (53.62)	458 (3.39)	13070 (96.61)	/	/	1 (ref)
Male	11703 (46.38)	518 (4.43)	11185 (95.57)	18.22	<0.0001	**1.32 (1.16-1.50)**
Age groups						
35-49	4240 (16.80)	60 (1.42)	4180 (98.58)	/	/	1 (ref)
50-59	8124 (32.20)	220 (0.87)	7904 (31.33)	30.38	<0.0001	**1.94 (1.45-2.59)**
60-69	9686 (38.39)	467 (4.82)	9219 (95.18)	8.17	0.0043	**3.53(2.69-4.63)**
70-75	2850 (11.30)	203 (7.12)	2647 (92.88)	59.76	<0.0001	**5.34(3.99-7.16)**
≥75	331 (1.31)	26 (7.85)	305 (92.15)	17.85	<0.0001	**5.94(3.70-9.55)**
Married						
No	1962 (7.78)	90 (4.59)	1872 (95.41)	/	/	1 (ref)
Yes	23269 (92.22)	886 (3.81)	22383 (96.19)	2.96	0.086	1.22 (0.97-1.52)
High school or above						
No	23736 (94.07)	929 (3.91)	22807 (96.09)	/	/	1 (ref)
Yes	1495 (5.93)	47 (3.14)	1448 (96.86)	2.38	0.14	1.26 (0.93-1.69)
Living in rural places						
No	6808 (26.98)	285 (4.19)	6523 (95.81)	/	/	1 (ref)
Yes	18423 (73.02)	691 (3.75)	17732 (96.25)	2.49	0.11	1.12 (0.97-1.29)
Household income is more than 50,000 yuan/year						
No	5015 (19.88)	153 (3.05)	4862 (96.95)	/	/	1 (ref)
Yes	20216 (80.12)	823 (4.07)	19393 (95.93)	11.25	0.0008	**1.35 (1.13-1.61)**
Smoking						
No	5020 (19.90)	157 (3.13)	4863 (96.87)	/	/	1 (ref)

Yes	20211 (80.10)	819 (4.05)	19392 (95.95)	9.34	0.007	**1.31 (1.10-1.56)**
Drinking						
Never	17297 (69.08)	767 (4.43)	16530 (95.57)	/	/	1 (ref)
More than 1/month	896 (3.58)	23 (2.57)	873 (97.43)	0.29	0.59	**0.57(0.37-0.86)**
2-3/month	1428 (5.70)	29 (2.03)	1399 (97.97)	4.23	0.04	**0.45(0.31-0.65)**
2-3/week	710 (2.83)	21 (2.96)	689 (97.04)	0.07	0.79	0.66(0.42-1.02)
More than 4/week	4709 (18.81)	122 (2.59)	4587 (97.41)	0.71	0.4	**0.57 (0.47-0.70)**
Overweight						
No	15555 (61.65)	575 (3.70)	14980 (96.30)	/	/	1 (ref)
Yes	9676 (38.35)	401 (4.14)	9275 (95.86)	3.19	0.07	1.13 (0.99-1.28)
Obesity						
No	4420 (17.52)	144 (3.26)	4276 (96.74)	/	/	1 (ref)
Yes	20811 (82.48)	832 (4.00)	19979 (96.00)	5.6	0.02	**1.24 (1.03-1.48)**
Waistline is large						
No	4423 (17.53)	85 (1.92)	4338 (98.08)	/	/	1 (ref)
Yes	20808 (82.47)	891 (4.28)	19917 (95.72)	64.26	<0.0001	**2.28 (1.82-2.86)**
Abnormal TG						
No	13778 (71.85)	655 (4.75)	13123 (95.25)	/	/	1 (ref)
Yes	5398 (28.15)	211 (3.91)	5187 (96.09)	6.61	0.01	**1.54 (1.41-1.68)**
Abnormal LDL						
No	18566 (73.58)	838 (4.51)	17728 (95.49)	/	/	1 (ref)
Yes	6665 (26.42)	138 (2.07)	6527 (97.93)	89.13	<0.0001	**1.86 (1.65-2.09)**
Abnormal TG						
No	12861 (50.97)	516 (4.01)	12345 (95.99)	/	/	1 (ref)
Yes	12370 (49.03)	460 (3.72)	11910 (96.28)	1.46	0.23	**1.07 (1.00-1.15)**
Abnormal HDL						

No	20950 (83.03)	828 (3.95)	20122 (96.05)	/	/	1 (ref)
Yes	4281 (16.97)	148 (3.46)	4133 (96.54)	2.41	0.12	1.15 (0.96-1.37)
Abnormal FPG						
No	14374 (74.76)	746 (5.19)	13628 (94.81)	/	/	1 (ref)
Yes	4852 (25.24)	98 (2.02)	4754 (97.98)	101.4	<0.0001	**2.13 (1.83-2.49)**
Action capability						
Normality	24896 (98.67)	910 (3.66)	23986 (96.34)	/	/	1 (ref)
Inconvenience	318 (1.26)	58 (18.24)	260 (81.76)	0.47	0.04	**5.88 (4.39-7.88)**
Bed-ridden	17 (0.07)	8 (47.06)	9 (52.94)	21.31	<0.0001	**23.45 (9.03-60.92)**
Self-care ability						
Yes	25096 (99.47)	946 (3.77)	24150 (96.23)	/	/	1 (ref)
In difficulties	116 (0.46)	23 (19.83)	93 (80.17)	2.18	0.14	**6.31 (3.98-10.01)**
No	19 (0.07)	7 (36.84)	12 (63.16)	13.19	0.0003	**14.89 (5.85-37.91)**
Daily activity ability						
Yes	24975 (98.98)	915 (3.66)	24060 (96.34)	/	/	1 (ref)
In difficulties	232 (0.92)	54 (23.28)	178 (76.72)	10.48	0.0012	**7.98 (5.84-10.89)**
No	24 (0.1)	7 (19.17)	17 (70.83)	8.68	0.0032	**10.83 (4.48-26.17)**
Pain/ Discomfort						
No	21138 (83.78)	754 (3.57)	20384 (96.43)	/	/	1 (ref)
Moderate	4053 (16.06)	218 (5.38)	3835 (94.62)	0.19	0.66	**1.53 (1.32-1.79)**
Extreme	40 (0.16)	4 (10.00)	36 (90.00)	2.8	0.09	**3.0 (1.07-8.46)**
Anxiety/depression/psychological stress						
No	23824 (94.42)	897 (3.77)	22927 (96.23)	/	/	1 (ref)
Moderate	1391 (5.52)	75 (5.39)	1316 (94.61)	148.9	0.026	**1.46 (1.14-1.86)**
Extreme	16 (0.06)	4 (25.00)	12 (75.00)	4.94	0.0008	**8.52 (2.74-26.47)**
History of hypertension						

No	13513 (53.56)	347 (2.57)	13166 (97.43)	/	/	1 (ref)
Yes	11718 (46.44)	629 (5.37)	11089 (94.63)	126.68	<0.0001	**2.15 (1.88-2.46)**
History of diabetes						
No	22975 (91.06)	831 (3.62)	22144 (96.38)	/	/	1 (ref)
Yes	2256 (8.94)	145 (6.43)	2111 (93.57)	42.41	<0.0001	**1.83 (1.53-2.20)**
History of dyslipidemia						
No	25189 (99.83)	971 (3.85)	24218 (96.15)	/	/	1 (ref)
Yes	42 (0.17)	5 (11.90)	37 (88.10)	6.57	0.011	**3.37 (1.32-8.60)**
A history of kidney or ureteral calculi						
No	25105 (99.50)	970 (3.86)	24135 (96.14)	/	/	1 (ref)
Yes	126 (0.50)	6 (4.76)	120 (95.24)	0.27	0.6	1.24 (0.55-2.83)
Family history of hypertension						
No	22351 (88.59)	801 (3.58)	21550 (96.42)	/	/	1 (ref)
Yes	2880 (11.41)	175 (6.08)	2705 (93.92)	41.63	<0.0001	**1.74 (1.47-2.06)**
Family history of coronary heart disease						
No	24591 (97.46)	943 (3.83)	23648 (96.17)	/	/	1 (ref)
Yes	640 (2.54)	33 (5.16)	607 (94.84)	2.93	0.087	1.37 (0.96-1.95)
Family history of ischemic stroke and cerebral infarction						
No	24763 (98.15)	943 (3.81)	23820 (96.19)	/	/	1 (ref)
Yes	468 (1.85)	33 (7.05)	435 (92.95)	12.56	0.0004	**1.92 (1.34-2.75)**
Family history of hemorrhagic stroke and cerebral hemorrhage						
No	24885 (98.63)	958 (3.85)	23927 (96.15)	/	/	1 (ref)
Yes	346 (1.37)	18 (5.20)	328 (94.80)	1.68	0.2	1.37 (0.85-2.21)

Family history of diabetes						
No	23796 (94.31)	930 (3.91)	22866 (96.09)	/	/	1 (ref)
Yes	1435 (5.69)	46 (3.21)	1389 (96.79)	1.79	0.18	0.81 (0.60-1.10)
Family history of hypercholesterolemia						
No	25152 (99.69)	976 (3.88)	24176 (96.12)	/	/	1 (ref)
Yes	79 (0.31)	0 (0.00)	79 (100.00)	0.0027	0.96	-
Hypoglycemic drugs use						
No	23541 (93.30)	865 (3.67)	22676 (96.33)	/	/	1 (ref)
Yes	1690 (6.70)	111 (6.57)	1579 (93.43)	34.47	<0.0001	**1.84 (1.50-2.26)**

Supplementary table 1. Baseline situation and related risk factors of CVD. FBG, Fasting plasma glucose; TC, Total cholesterol; TG, triglycerides; LDL, Low density lipoprotein; HDL, High density lipoprotein; Sociodemographic characteristics of participants were summarized by areas using frequencies (percentages), and they were compared by Chi-squared tests, and data were analyzed using Yates's continuity correction or Fisher's exact probability test as necessary. Univariate logistic regression model was performed to identify independent risk factors. Statistically significant results are presented in bold.
